# The Possible Role of Mena Protein and Its Splicing-Derived Variants in Embryogenesis, Carcinogenesis, and Tumor Invasion: A Systematic Review of the Literature

**DOI:** 10.1155/2013/365192

**Published:** 2013-07-14

**Authors:** Simona Gurzu, Diana Ciortea, Istvan Ember, Ioan Jung

**Affiliations:** ^1^Department of Pathology, University of Medicine and Pharmacy of Targu-Mures, 38 Ghe Marinescu Street, 540193 Targu Mures, Romania; ^2^Department of Dermatology, University of Medicine and Pharmacy of Targu-Mures, 540193 Targu Mures, Romania; ^3^Department of Public Health, University of Pecs, Medical School, Szigeti Street 12, Pecs 7624, Hungary

## Abstract

The Ena/VASP (enabled/vasodilator stimulated phosphoprotein) family includes the binding actin proteins such as mammalian Ena (Mena), VASP, and Ena-VASP-like. It is known that the perturbation of actin cycle could determine alteration in the mobility of cells and in consequence of organogenesis. Few recent studies have revealed that Mena protein could play a role in breast or pancreatic carcinogenesis. Based on our researches, we observed that the intensity of Mena expression increased from premalignant to malignant lesions in some organs such as large bowel, stomach, cervix, and salivary glands. These findings prove that Mena could be a marker of premalignant epithelial lesions. In premalignant lesions, it could be helpful to define more accurately the risk for malignant transformation. In malignant tumors, correlation of expression of its splice variants could indicate metastatic behavior. In conclusion, we consider that it is necessary to analyze the expression of Mena splice variants in a higher number of cases, in different epithelial lesions, and also in experimental studies to define its exact role in carcinogenesis and also its possible prognostic and predictive values.

## 1. Introduction

First data about Mena (mammalian Ena homolog) protein, a relative of VASP (vasodilator stimulated phosphoprotein) and *Drosophila* protein enabled (Ena), were published in 1996 by Gertler et al. [1]. Since then, about 60 studies have been added, most of them presenting the results of *in vivo* or *in vitro* experiments. In this review, we intended to correlate our original results related to the possible prognostic and predictive roles of this protein in clinical oncology with the literature data.

## 2. Basic Information about Mena Protein and Its Splicing Variants 

Mena is a protein involved in the nucleation and polymerization of actin, being a cellular regulator of assembling and dynamic of cytoplasmic actin networks [2, 3]. It belongs to the Ena/VASP (enabled/VASP) family, along with VASP and Ena-VASP-like, being encoded by Mena gene located on chromosome 1 [4]. It was first identified in mouse, when other splicing-derived isoforms of pan-Mena were described: a human homologue (pan-hMena), a variant present in primary tumor cells but lost in invasive cells (Mena^11a^), two invasive forms (Mena^INV^ or Mena^++^ and Mena^+++^), and the subtype MenaΔv6 [1, 2, 4–8]. These isoforms seem to be partially tissue-specific and are partially classified based on their function [4, 9]. The differences are mainly related to the number of exons as follows: neuronal variant presents an extended exon 6, the spleen variant is characterized by the absence of proline-rich region, and the breast variant has a supplementary exon 11a [1, 2, 4, 10]. On the other hand, when compared with the 570-amino acids pan-hMena, the isoforms Mena^INV^ (+++) and Mena^++^ contain a supplementary exon, next to the Ena/VASP homology 1 (EVH1) domain (a 4-amino acid region for ++ and a 19-amino acid region for +++); the variant hMena^11a^ contains the exon 11a included within the EVH2 domain, adjacent to the F-actin motif. In contrast, hMenaΔv6 lacks the internal exon 6 [1, 2, 4–6, 9, 10]. 

Ena/VASP proteins were identified in *Listeria monocytogenes, Shigella flexneri,* and *Dictyostelium* that uses the actin cytoskeleton from their host [11, 12]. In mouse tissues, Mena expression was observed in brain, gastrointestinal tract, blood vessels, mesangial membrane of the kidney, and also in the membrane of epithelial cells. Its expression decreased from embryonic to adult life and was not observed in platelets and spleen [13].

There are studies that have revealed that Ena/VASP proteins are involved in those processes where dynamic actin reorganization is necessary, such as neural closure, phagocytosis, hemostasis, chemotaxis, cell migration, cell-cell and cell-matrix adhesions, fibrillogenesis, or fibroblastic motility [14–16]. However, the exact mechanism of their activity is not yet known.

The instability of actin cycle could determine aberrant cell motility and adhesion. The alteration of mobility cells via actin polymerization could be determined by actin mutation or Ena/VASP alterations. In the embryonic life, the consequence could be disorder of organogenesis, but, in adult life, it could promote malignant transformation, tumor cell invasion, or metastatic spread [16–18]. 

Most of the published studies are experimental, with breast cell cultures being commonly used. Mena activity could also be quantified in the cytoplasm of the tumor cells using immunohistochemical reactions, with the internal control being the smooth muscle cells or human cerebellum. While antibodies to the pan-Mena and 11a are available to assess their relevance *in vivo*; antibodies to *Δv6 *are not available, and, thus, its expression is arbitrarily assumed on the basis of the pan-Mena^+^/11a phenotype.

Molecular mRNA examinations can also be carried out to determine the genetic mutations of Mena. We have described for the first time the immunohistochemical aspects of Mena protein in cervical, colorectal, gastric lesions, and also lesions of the salivary glands [19–22]. In addition, they have been further depicted in correlation with the literature data. 

## 3. Mena Expression in Nontumor, Premalignant, and Malignant Lesions 

### 3.1. Mammary Gland Development, Breast Lesions, and Culture Cell Lines

Roussos et al. observed that during postnatal mammary gland development, Mena deficiency does not affect duct length, but the outgrowth of terminal end buds and ductal branching are delayed [7]. Similar to other organs, Mena activity decreases from embryonal to adult life, but it can be reloaded in breast tumors. The first data about Mena activity related to breast cancer were reported by Di Modugno et al. in 2004 [4]. They observed that the pan-hMena was immunohistochemically expressed in about 70% of primary breast carcinomas, 90% of metastatic carcinomas, and also in 67% of the breast lesions with a high risk for malignant transformation (atypical papilloma, hyperplasia) [5], when compared with only 9% in low-risk benign diseases such as adenosis, ductal ectasia, cysts, fibroadenomas, and simple apocrine metaplasia [8]. These authors revealed that, in breast lesions, pan-hMena expression increased during malignant transformation and played an important role in tumor invasiveness. Upregulation of pan-hMena intensity was observed in the carcinomas that expressed the immunophenotype HER-2+/ER-/PR-/Ki67+ and was correlated with tumor size, advanced clinical stages, and highly invasive properties [5, 8]. Mena positivity was also noted in the synchronous or metachronous metastases (lymph nodes, lung, brain, and pleura), and a juxtamembrane reinforcement was observed in these metastatic lesions [8]. These observations were partially confirmed by Du et al. using an immunohistochemical study; they added that Mena intensity does not depend on the histological type of HER-2-positive breast carcinomas and can also be observed in about 7% of normal breast tissue [23], although Di Modugno et al. denied detection of hMena isoforms in normal breast tissue [5, 8]. Technical differences and different types of specimen conservations could explain these conflicting results. However, the HER-2-Mena relationship, agreed by both groups [5, 23], is also sustained by downregulation of hMena intensity in patients treated with the anti-HER-2 drug named Herceptin [4, 8].

hMena activity, as a cytoskeleton remodeling protein, also seems to be strongly related to E-cadherin, but E-cadherin expression and Ki67 positivity are noted only in hMena-positive/hMena^11a^-positive cases, when compared with hMena-positive/hMena^11a^-negative ones [5]. hMena^11a^ is also phosphorylated downstream of HER-2 and EGF (epidermal growth factor), and Mena^INV^ facilitates invasion of tumor cells and their discohesive morphology [5]. *In vivo, *Mena deficiency decreases EGF-induced invasion and motility [7]. However, the two splice variants are mutually exclusive, and the 11a variant inhibits the expression of Mena^INV^. An accelerated tumor cell invasion as a result of PDGF (platelet-derived growth factor) and Mena interaction in a phosphatidylinositol-3-kinase (PI3K)-dependent manner has also been hypothesized [24] similar to a macrophage-mediated invasion [7]. 

The isoform 11a is expressed in both premalignant and malignant cells, and, during tumor progression, it seems to be downregulated; at the same time, a tumor-environment-dependent switching pattern occurs, and the pan-Mena isoforms ++ and +++ become upregulated when tumor cells become invasive *in vivo*, in both human and mice [6]. According to Goswami et al., the pan-Mena splice variants ++ and +++ remained upregulated in blood flow and also in the tumor cells that formed lung metastases; no expression of 11a variant was identified in the metastatic cells, except in the culture cell lines treated with EGF [6]. On the other hand, Mena deficiency was found to reduce the speed of tumor cell invasion, intravasation, and motility, although tumor burden and growth were noted to remain unaffected; as a result, it was concluded that Mena inhibition delays tumor progression and metastatic dissemination but cannot prevent progression to malignancy [7]. 

One of the newest and very interesting experiments revealed that, in cultures of invasive mesenchymal breast cancer cells, induction of mesenchymal-to-epithelial transition produced a reexpression of hMena^11a^, modification of cytoskeletal architecture, and decrease in cell invasiveness [5].

The meaning of these new findings related to splice variants of Mena protein should be explored in the further studies.

### 3.2. Colorectal and Gastric Lesions

Our previous results showed that Mena was not immunohistochemically expressed in the normal colorectal mucosa and in the adenomatous polyps without dysplasia; however, in polyps with high dysplasia, Mena was overexpressed. In colorectal carcinomas, Mena marked the tumor cells in 80% of the cases, and the results were later confirmed by Toyoda et al. [25]. In 25% of positive cases, the intensity was very high (3+); in 60%, it was moderate (2+); and in the other 15%, it was low-expressed (1+). Moreover, Mena intensity was higher in the microsatellite stable tumors (MSS), when compared with those with microsatellite instability, and was correlated with vascular invasion, tumor budding, tumor stage, intensity of angiogenesis marked with CD31 and CD105, and with HER-2, and p53 expression [19, 25]. These observations are in agreement with those obtained in the case of breast cancer [5, 23], confirming Mena-HER-2 interaction in the tumors of the gastrointestinal tract as well. In contrast to colorectal cancer, Mena intensity does not seem to affect the vascular density in breast cancer, although vascular invasion is strongly influenced [7]. A possible pathway in these tumors can be related to Wnt/*β*-catenin, as Najafov et al. proved it experimentally in colorectal, breast, and hepatocellular carcinoma cell lines [26]. 

We also observed an increasing intensity of Mena expression from benign to malignant lesions of the stomach, but the results were published only in abstract [20]. 

### 3.3. Pancreatic Lesions

There is only one study that revealed the overexpression of Mena in primary and metastatic pancreatic carcinomas [27]. In agreement with the observations by Di Modugno et al. in cases of breast cancer lines [4], Pino et al. revealed that EGFR played an important role in the phosphorylation of hMena^11a^ [27]. The 11a variant was exclusively detected in the pancreatic cell lines that also expressed E-cadherin positivity, which was not found in E-cadherin negative cells [6, 27].

### 3.4. Premalignant and Malignant Lesions of Cervix

In our study published in 2009, which is the only study in the literature related to cervical lesions, Mena immunostain was not observed in the normal cervical squamous epithelium; however, its intensity was increased in parallel with the grade of CIN (cervical intraepithelial lesion). In the cases with low-grade CIN, Mena was only expressed in the basal parts of squamous epithelium and extended through the full thickness of the epithelium in the case of *in situ* cervical squamous cell carcinomas. All invasive cervical squamous cell carcinomas presented overexpression of Mena protein [21].

### 3.5. Lesions of the Salivary Glands

In our most recent study published in 2012, we revealed Mena expression in carcinomas of the salivary glands and its correlation with the tumor grade, confirming the possible role of Mena in carcinogenesis and aggressivity, similar to breast, cervical, colorectal, and gastric carcinomas. Another particular aspect observed in our cases was Mena positivity in lymphoblasts and endothelial cells but no expression in lymphoma cells [22]. 

### 3.6. Dilated Cardiomyopathy and Wound Healing

As regulators of cytoplasmic actin dynamics, Ena/VASP proteins seem to have an essential role in wound healing [18], and a two-fold increase in cell motility has been reported in a wound-healing *in vivo* experiment, as a result of Mena stimulation [28]. This effect could be beneficial in case of myocardial infarction. 

On the other hand, displacement of Ena/VASP proteins from cardiac intercalated disks during embryogenesis, determined in one of the *in vivo* experiments performed on transgenic mice, resulted in severe dilated cardiomyopathy with dilatation of all cardiac chambers, myocyte hypertrophy, bradycardia, and early postnatal lethality [29], although the cardiac abnormalities were initially denied by Gertler et al. [1]. Similar to studies related to breast tumor cell cultures, in case of cardiac embryogenesis, VASP, Mena, and E-cadherin were colocalized in the intercalated disk, and the authors suggested that Mena could not only be implied in actin remodeling but also in intercellular communication [24, 29]. This role was also proved in the adult knockout mice Mena−/− that presented low levels of vinculin, another adherence junction protein, when compared with the wild-type mice [30]. 

Cardiac failure with prolonged electrocardiogram PR and QRS intervals, coupled with myocytes hypertrophy and cardiac fibrosis in the case of genetic ablation of Mena, was also reported [30]. In addition, Bear et al. proved that deletion of both Mena and VASP genes during late embryonic development was lethal in mice [29, 31]. 

### 3.7. Embryogenesis/Tumorigenesis of the Central Nervous System and Alteration of Noise Sensitivity

Axonal morphogenesis is one of the embryonic processes in which Mena activity was experimentally analyzed. A functional nervous system requires adequate cell motility, and, as a consequence, a proper recruitment of Ena/VASP proteins is mandatory to regulate the leading edge of cells [18]. Similar to studies related to breast tumor cell cultures, in case of axonal embryogenesis, it was proved that normal Mena phosphorylation requires PDGF stimulation [18, 24]. 

Besides axonal disorders, Mena−/− mice presented hippocampal commissure defects, and the VASP−/− ones exhibited megakaryocyte hyperplasia in bone marrow and spleen as well as increased platelets activity [32]; the latter confirmed the possible Mena/VASP-PDGF interactions. In case of mice with neural tube closure defects, such as anencephaly and spina bifida, the genes involved in actin remodeling, including Mena gene, were detected to be present mutations [33]. At the same time, the commissure formation is regulated through Wnt/*β*-catenin pathway, with the Wnt4 and Wnt5 being implicated and lithium-induced inhibition of *β*-catenin degradation resulting in increased Mena transcription [26]. Mena deficiency via Wnt/*β*-catenin pathway disorders seems to be related to both dysregulation of embryogenesis as well as susceptibility to schizophrenia [26, 34].

In 2004, an experiment performed by Schick et al. in VASP−/− mice revealed that VASP and Mena share similar cellular localization and functions. In their experiment, when compared with wild-type mice, the VASP−/− ones presented increased noise sensitivity at lower frequencies; however, no differences in auditory brainstem responses have been observed. Mena was identified in the cochlear cells of the wild-type mice but not in the VASP−/− ones [32]. The significance of this experiment should be further studied.

With regard to brain tumors, one of the published studies took into account the role of Mena protein in glioblastoma cell lines. Higashi et al. proved that *in vivo* siRNA Mena depletion increased tumor cell motility via suppression of Rac1 activity, and this protein is a member of the Rho family GTPases [35].

### 3.8. Last but Not the Least, the Kidney Podocyte

Presence of Mena expression in mesangial membrane of the mouse kidney, with important decreasing level from embryonic to adult life, reported in 2001 [13], was later confirmed in 2007, and Yanagida-Asanuma et al. proved that proper function and structure of the actin-based foot processes of kidney podocytes are strongly related to complex Cdc42:IRSp53:Mena. Disorders in its assembling can be performed using synaptopodin, a proline-rich actin-associated protein that blocks Mena binding; the result is lipopolysaccharide-induced proteinuria [36]. Interestingly, synaptopodin was also identified in brain tissue, and its absence was associated with deficits in activity-dependent synaptic plasticity [36, 37]. 

## 4. Possible Predictive Role of Mena Protein in Tumors and Other Lesions

In case of pancreatic cancer, based on the direct correlation between Mena and EGFR, Pino et al. suggested that Mena expression could be a predictive factor of EGFR inhibitors such as Erlotinib, and this orally administrated drug is already used in combination with gemcitabine in patients with advanced pancreatic carcinomas [27]. Takahashi and Suzuki added that depletion of Mena by RNA interference abrogated both cell invasion and actin accumulation at the invasion site, stopping tumor cell invasion in breast cells culture [24]. Although its inhibition cannot block the metastatic process or decrease the vascular density, the functional intervention targeting Mena in breast cancer patients could delay tumor progression and invasion, which can represent a valuable future therapy independent of tumor size [7]. In tumors of gastrointestinal tract, Mena depletion could be realized using substances that act through the Wnt/*β*-catenin pathway [26].

The most concrete aspect regarding the possible predictive value of Mena protein is related to TES gene, a Mena interacting partner [38–40]. TES tumor suppressor gene was first described in 2001 as a gene located on the human chromosome 7q31.1 that encodes a 421-amino acid protein localized in the cell-cell contact areas, with a role in focal cellular adhesion; its upregulation reduced the tumor growth in case of ovarian and cervical carcinoma cell lines [38, 39]. To produce actin assembly, TES interacts with zyxin, similar to Mena protein [39], but not with VASP protein [40], although VASP and Mena seem to share similar cellular localization and functions [31]. Based on these observations, Boëda et al. suggested that TES can be attached to Mena and block its interactions, resulting in inhibition of tumor cells invasion in a Mena-dependent mechanism [40].

According to Yanagida-Asanuma et al., Mena depletion could also be used as a new antiproteinuric target [36].

As the levels of Mena should be increased to accelerate wound healing [18, 28], lithium-induced *β*-catenin increases Mena transcription in these cases [26]. 

## 5. Summary and Perspectives

The new findings presented in this review prove that Mena could be a new marker for different premalignant epithelial lesions and tumors. It is difficult to define its exact role in embryogenesis and tumorigenesis, but it seems that the intensity of pan-Mena antibody increases from benign to malignant lesions. The pan-Mena immunostain associated with Mena^11a^ expression could help the pathologist to define more accurately the risk for malignant transformation in some lesions such as cervical dysplasia. 

This could have therapeutic impact and could change the molecular classification of breast cancers. At the same time, genetic counseling could include detection of Mena mutations in case of suspicion for tube closure defects, renal disorders, or cardiomyopathy.

The correlation between Mena intensity and microvascular density in colorectal carcinomas showed that Mena intensity in the tumor cells could be used to predict response to antiangiogenic treatment in case of metastatic colorectal carcinomas. Downregulation of Mena^11a^ splice variant associated with upregulation of its invasive counterpart Mena^INV^ could predict the risk of metastasis. However, as the tumor microenvironment cannot be totally reproduced *in vivo*, the real clinical impact of these determinations is still an enigma.

Several antibodies have been discovered throughout the years, and many researchers believe that the life expectancy could be prolonged if some drugs could be synthesized based on their expression. Probably, Mena is one of these too many antibodies or probably not. If the real meaning of the Mena protein and its splicing-derived isoforms could be correctly defined, the pathologists, clinicians, and patients could have real benefits.

## References

[1] Gertler FB, Niebuhr K, Reinhard M, Wehland J, Soriano P (1996). Mena, a relative of VASP and Drosophila enabled, is implicated in the control of microfilament dynamics. *Cell*.

[2] Philippar U, Roussos ET, Oser M (2008). A Mena invasion isoform potentiates EGF-induced carcinoma cell invasion and metastasis. *Developmental Cell*.

[3] Krause M, Dent EW, Bear JE, Loureiro JJ, Gertler FB (2003). Ena/VASP proteins: regulators of the actin cytoskeleton and cell migration. *Annual Review of Cell and Developmental Biology*.

[4] Di Modugno F, Bronzi G, Scanlan MJ (2004). Human Mena protein, a serex-defined antigen overexpressed in breast cancer eliciting both humoral and CD8^+^ T-cell immune response. *International Journal of Cancer*.

[5] Di Modungo F, Iapicca P, Boudreau A (2012). Splicing program of human Mena produces a previously undescribed isoform associated with invasive, mesenchymal-like breast tumors. *Proceedings of the National Academy of Sciences of the United States of America*.

[6] Goswami S, Philippar U, Sun D (2009). Identification of invasion specific splice variants of the cytoskeletal protein Mena present in mammary tumor cells during invasion in vivo. *Clinical and Experimental Metastasis*.

[7] Roussos ET, Wang Y, Wyckoff JB (2010). Mena deficiency delays tumor progression and decreases metastasis in polyoma middle-T transgenic mouse mammary tumors. *Breast Cancer Research*.

[8] Di Modugno F, Mottolese M, Di Benedetto A (2006). The cytoskeleton regulatory protein hMena (ENAH) is overexpressed in human benign breast lesions with high risk of transformation and human epidermal growth factor receptor-2-positive/hormonal receptor-negative tumors. *Clinical Cancer Research*.

[9] Di Modugno F, DeMonte L, Balsamo M (2007). Molecular cloning of hMena (ENAH) and its splice variant hMena^+11a^: epidermal growth factor increases their expression and stimulates hMena^+11a^ phosphorylation in breast cancer cell lines. *Cancer Research*.

[10] Tani K, Sato S, Sukezane T (2003). Abl interactor 1 promotes tyrosine 296 phosphorylation of mammalian enabled (Mena) by c-ABL kinase. *The Journal of Biological Chemistry*.

[11] Kwiatkowski AV, Gertler FB, Loureiro JJ (2003). Function and regulation of Ena/VASP proteins. *Trends in Cell Biology*.

[12] Higley S, Way M (1997). Actin and cell pathogenesis. *Current Opinion in Cell Biology*.

[13] Gambaryan S, Hauser W, Kobsar A, Glazova M, Walter U (2001). Distribution, cellular localization, and postnatal development of VASP and Mena expression in mouse tissues. *Histochemistry and Cell Biology*.

[14] Pula G, Krause M, Klussmann E, Scott J (2008). Role of Ena/VASP Proteins in Homeostasis and Disease. *Protein-Protein Interactions as a New Drug Targets*.

[15] Cardinali G, Kovacs D, Mastrofrancesco A (2013). hMena: altered expression in psoriatic skin. *Archives of Dermatological Research*.

[16] Gupton SL, Riquelme D, Hughes-Alford SK (2012). Mena binds *α*5 integrin directly and modulates *α*5*β*1 function. *Journal of Cell Biology*.

[17] Lambrechts A, van Troys M, Ampe C (2004). The actin cytoskeleton in normal and pathological cell motility. *International Journal of Biochemistry and Cell Biology*.

[18] Michael M, Vehlow A, Navarro C, Krause M (2010). c-Abl, Lamellipodin, and Ena/VASP proteins cooperate in dorsal ruffling of fibroblasts and axonal morphogenesis. *Current Biology*.

[19] Gurzu S, Jung I, Prantner I, Ember I, Pávai Z, Mezei T (2008). The expression of cytoskeleton regulatory protein Mena in colorectal lesions. *Romanian Journal of Morphology and Embryology*.

[20] Jung I, Gurzu S, Prantner I, Mezei T, Pavai Z (2008). Immunohistochemical expression of Mena in carcinomas and premalignant lesions of colon, stomach and thyroid gland. *Histopathology*.

[21] Gurzu S, Jung I, Prantner I, Chira L, Ember I (2008). The immunohistochemical aspects of protein Mena in cervical lesions. *Romanian Journal of Morphology and Embryology*.

[22] Gurzu S, Ember I, Krause M (2012). Mena, a new available marker in tumors of salivary glands?. *European Journal of Histochemistry*.

[23] Du JW, Xu KY, Fang LY, Qi XL (2012). Clinical significance of Mena and Her-2 expression in breast cancer. *European Journal of Gynaecological Oncology*.

[24] Takahashi K, Suzuki K (2011). WAVE2, N-WASP, and Mena facilitate cell invasion via phosphatidylinositol 3-kinase-dependent local accumulation of actin filaments. *Journal of Cellular Biochemistry*.

[25] Toyoda A, Kawana H, Azuhata K (2009). Aberrant expression of human ortholog of mammalian enabled (hMena) in human colorectal carcinomas: implications for its role in tumor progression. *International Journal of Oncology*.

[26] Najafov A, Seker T, Even I (2012). MENA is a transcriptional target of the Wnt/Beta-catenin pathway. *PLoS ONE*.

[27] Pino MS, Balsamo M, Modugno FD (2008). Human Mena^+11a^ isoform serves as a marker of epithelial phenotype and sensitivity to epidermal growth factor receptor inhibition in human pancreatic cancer cell lines. *Clinical Cancer Research*.

[28] Peng Y, Murray EL, Sarkar M, Liu X, Schoenberg DR (2009). The cytoskeleton-associated Ena/VASP proteins are unanticipated partners of the PMR1 mRNA endonuclease. *RNA*.

[29] Eigenthaler M, Engelhardt S, Schinke B (2003). Disruption of cardiac Ena-VASP protein localization in intercalated disks causes dilated cardiomyopathy. *The American Journal of Physiology*.

[30] Aguilar F, Belmonte SL, Ram R (2011). Mammalian enabled (Mena) is a critical regulator of cardiac function. *The American Journal of Physiology*.

[31] Bear JE, Loureiro JJ, Libova I, Fässler R, Wehland J, Gertler FB (2000). Negative regulation of fibroblast motility by Ena/VASP proteins. *Cell*.

[32] Schick B, Praetorius M, Eigenthaler M (2004). Increased noise sensitivity and altered inner ear MENA distribution in VASP −/− mice. *Cell and Tissue Research*.

[33] Juriloff DM, Harris MJ (2000). Mouse models for neural tube closure defects. *Human Molecular Genetics*.

[34] Katsu T, Ujike H, Nakano T (2003). The human frizzled-3 (FZD3) gene on chromosome 8p21, a receptor gene for Wnt ligands, is associated with the susceptibility to schizophrenia. *Neuroscience Letters*.

[35] Higashi M, Ishikawa C, Yu J (2009). Human mena associates with Rac1 small GTPase in glioblastoma cell lines. *PLoS ONE*.

[36] Yanagida-Asanuma E, Asanuma K, Kim K (2007). Synaptopodin protects against proteinuria by disrupting Cdc42:IRSp53:Mena signaling complexes in kidney podocytes. *The American Journal of Pathology*.

[37] Deller T, Korte M, Chabanis S (2003). Synaptopodin-deficient mice lack a spine apparatus and show deficits in synaptic plasticity. *Proceedings of the National Academy of Sciences of the United States of America*.

[38] Tobias ES, Hurlstone AFL, MacKenzie E, Mcfarlane R, Black DM (2001). The TES gene at 7q31.1 is methylated in tumours and encodes a novel growth-suppressing LIM domain protein. *Oncogene*.

[39] Coutts AS, MacKenzie E, Griffith E, Black DM (2003). TES is a novel focal adhesion protein with a role in cell spreading. *Journal of Cell Science*.

[40] Boëda B, Briggs DC, Higgins T (2007). Tes, a specific Mena interacting partner, breaks the rules for EVH1 binding. *Molecular Cell*.

